# Supplementary material for: Impact of economic globalisation on value-added agriculture, globally
Source: PLoS One. 2023 Jul 21;18(7):e0289128. doi: 10.1371/journal.pone.0289128 (PMC10361532; doi:10.1371/journal.pone.0289128)
Supplement: S2 Appendix — (DOCX) [file pone.0289128.s002.docx]

**S2 Appendix. Specification test results for global and different income groups**

**S2.1 Appendix. Specification test results for global (All countries)**

| **All Countries** | **Tests** | | |
| --- | --- | --- | --- |
|  | **F test** | **LM Test** | **Hausman Test (Sigmamore)** |
|  | **H_0_: POLS** | **H_0_: POLS** | **H_0_: Random Effect** |
|  | **H_1_: Fixed Effect** | **H_1_: Random Effect** | **H_1_: Fixed Effect** |
| Y =f (EA) | 5036.77*** | 14369.21*** | 34.62*** |
| Y = f (EA ARMI) | 2675.73*** | 14283.08*** | 35.38*** |
| Y = f (EA ARMI FC) | 1868.63*** | 14281.56*** | 35.10*** |
| Y = f (EA ARMI FC FDI) | 1416.08*** | 14268.36*** | 35.05*** |
| Y= f (EA ARMI FC FDI ER) | 1220.74*** | 14306.92*** | 31.35*** |

Note: The symbols *, **and *** represents 10%, 5% and 1% significance level, respectively

**S2.2 Appendix. Specification test results for high income countries**

| **High-Income Level** | **Tests** | | |
| --- | --- | --- | --- |
|  | **F test** | **LM Test** | **Hausman Test (Sigmamore)** |
|  | **H_0_: POLS** | **H_0_: POLS** | **H_0_: Random Effect** |
|  | **H_1_: Fixed Effect** | **H_1_: Random Effect** | **H_1_: Fixed Effect** |
| Y =f (EA) | 479.48*** | 4717.83*** | 0.01 |
| Y =f (EA FDI) | 241.29*** | 4710.24*** | 0.15 |
| Y = f (EA FDI ER) | 206.07*** | 4667.96*** | 0.39 |
| Y = f (EA FDI ER ARMI) | 165.43*** | 4536.99*** | 0.75 |
| Y = f (EA FDI ER ARMI FC) | 137.96*** | 4534.82*** | 0.74 |

Note: The symbols *, **and *** represents 10%, 5% and 1% significance level, respectively

**S2.3 Appendix. Specification test results for low-income countries**

| **Low-Income Level** | **Tests** | | |
| --- | --- | --- | --- |
|  | **F test** | **LM Test** | **Hausman Test (Sigmamore)** |
|  | **H_0_: POLS** | **H_0_: POLS** | **H_0_: Random Effect** |
|  | **H_1_: Fixed Effect** | **H_1_: Random Effect** | **H_1_: Fixed Effect** |
| Y = f (FC) | 96.71*** | 1070.77*** | 2.13 |
| Y = f (FC, EA) | 74.81*** | 973.12*** | 4.08 |
| Y = f (FC, EA, ER) | 49.41*** | 959.10*** | 5.86 |
| Y = f (FC, EA, ER, ARMI) | 37.01*** | 962.21*** | 5.55 |
| Y = f (FC, EA, ER, ARMI, FDI) | 33.05*** | 721.21*** | 14.96*** |

Note: The symbols *, **and *** represents 10%, 5% and 1% significance level, respectively

**S2.4 Appendix. Specification test results for lower-middle income countries**

| **Lower-Middle Income** | **Tests** | | |
| --- | --- | --- | --- |
|  | **F test** | **LM Test** | **Hausman Test (Sigmamore)** |
|  | **H_0_: POLS** | **H_0_: POLS** | **H_0_: Random Effect** |
|  | **H_1_: Fixed Effect** | **H_1_: Random Effect** | **H_1_: Fixed Effect** |
| Y = f (EA) | 709.37*** | 3322.83*** | 3.48* |
| Y = f (EA FDI) | 352.75*** | 3210.15*** | 5.87* |
| Y = f (EA FDI FC) | 246.67*** | 3020.09*** | 8.01** |
| Y = f (EA FDI FC ARME) | 206.31*** | 2819.40*** | 8.36* |
| Y = f (EA FDI FC ARME ER) | 165.84*** | 2830.01*** | 9.67* |

Note: The symbols *, **and *** represents 10%, 5% and 1% significance level, respectively

**S2.5 Appendix. Specification test results for upper-middle income countries**

| **Upper-Middle Income** | **Tests** | | |
| --- | --- | --- | --- |
|  | **F test** | **LM Test** | **Hausman Test (Sigmamore)** |
|  | **H_0_: POLS** | **H_0_: POLS** | **H_0_: Random Effect** |
|  | **H_1_: Fixed Effect** | **H_1_: Random Effect** | **H_1_: Fixed Effect** |
| Y = f(EA) | 257.73*** | 2409.06*** | 10.29*** |
| Y = f(EA ARME ) | 178.71*** | 2409.75*** | 7.93** |
| Y = f(EA ARME ARMI) | 119.81*** | 2428.38*** | 6.82* |
| Y = f(EA ARME ARMI ER) | 117.70*** | 2290.74*** | 7.85* |
| Y = f(EA ARME ARMI ER FDI) | 94.21*** | 2285.20*** | 7.92 |
| Y = f(EA ARME ARMI ER FDI FC) | 86.24*** | 2157.79*** | 9.06 |

Note: The symbols *, **and *** represents 10%, 5% and 1% significance level, respectively
