# Supplementary material for: Impact of economic globalisation on value-added agriculture, globally
Source: PLoS One. 2023 Jul 21;18(7):e0289128. doi: 10.1371/journal.pone.0289128 (PMC10361532; doi:10.1371/journal.pone.0289128)
Supplement: S3 Appendix — (DOCX) [file pone.0289128.s003.docx]

| **All Countries** | | | | | | | | | | |
| --- | --- | --- | --- | --- | --- | --- | --- | --- | --- | --- |
| **Equation** | **Y = f (EA)** | | **Y= f (EA ARMI)** | | **Y = f (EA ARMI FC)** | | **Y = f (EA ARMI FC FDI** | | **Y= f (EA ARMI FC FDI ER)** | |
| **Variables** | **AVA** | | **AVA** | | **AVA** | | **AVA** | | **AVA** | |
|  | **FE** | **RE** | **FE** | **RE** | **FE** | **RE** | **FE** | **RE** | **FE** | **RE** |
| **FC** |  |  |  |  | -7.03e-06 | -0.0000139 | -6.93e-06 | -0.0000138 | -6.79e-06 | -0.0000133 |
|  |  |  |  |  | (0.0000104) | (0.0000126) | (0.0000105) | (0.0000127) | (.0000104) | (0.0000126) |
| **EA** | 0.2566*** | 0.2793*** | 0.2558*** | 0.2787*** | 0.2557*** | 0.2784*** | 0.2556*** | 0.2783*** | 0.2616*** | 0.2825*** |
|  | (0.0456) | (0.0386) | (0.0466) | (0.0393) | (0.0466) | (0.0394) | (0.0466) | (0.0394) | (0.0476) | (0.0400) |
| **ARME** |  |  |  |  |  |  |  |  |  |  |
|  |  |  |  |  |  |  |  |  |  |  |
| **ARMI** |  |  | 0.03679*** | 0.0264*** | 0.0367 | 0.0263 | 0.0368 | 0.0265 | 0.0029 | -0.00983 |
|  |  |  | (0.1477) | (0.1407) | (0.1477) | (0.1407) | (0.1477) | (0.1408) | (0.1416) | (0.1344) |
| **FDI** |  |  |  |  |  |  | 0.0012 | 0.00090 | 0.0012 | 0.000942 |
|  |  |  |  |  |  |  | (0.0019) | (0.0019) | (0.0019) | (0.00192) |
| **ER** |  |  |  |  |  |  |  |  | 0.000143*** | 0.00015*** |
|  |  |  |  |  |  |  |  |  | (0.0000544) | (0.0000471) |
| **Constant** | 3.9609 | 3.3939 | 3.9287 | 3.3703 | 3.9307 | 3.3791 | 3.9248 | 3.3783 | 3.7150 | 3.2086 |
| **No of Countries** | 101 | 101 | 101 | 101 | 101 | 101 | 101 | 101 | 101 | 101 |
| **No of years** | 22 | 22 | 22 | 22 | 22 | 22 | 22 | 22 | 22 | 22 |
| **R^2^ Within** | 0.2787 | 0.2787 | 0.2788 | 0.2788 | 0.2788 | 0.2788 | 0.2788 | 0.2788 | 0.2865 | 0.2864 |
| **R^2^ Between** | 0.8033 | 0.8033 | 0.8036 | 0.8035 | 0.8036 | 0.8035 | 0.8035 | 0.8034 | 0.7988 | 0.7990 |
| **R^2^ Overall** | 0.7673 | 0.7673 | 0.7676 | 0.7675 | 0.7676 | 0.7675 | 0.7675 | 0.7675 | 0.7643 | 0.7645 |

**S3 Appendix. Results of panel regression for global and different income groups**

**S3.1 Appendix. Panel regression for global (All countries)**

Note: The symbols *, **, and *** represents 10%, 5%, and 1% significance level, respectively.

Parentheses represent the robust standard error. FE and RE represent the Fixed effect and Random effect, respectively.

| **High-Income Level** | | | | | | | | | | |
| --- | --- | --- | --- | --- | --- | --- | --- | --- | --- | --- |
| **Equation** | **Y=f (EA)** | | **Y=f (EA FDI)** | | **Y = f (EA FDI ER)** | | **Y = f (EA FDI ER ARMI)** | | **Y = f (EA FDI ER ARMI FC)** | |
| **Variables** | **AVA** | | **AVA** | | **AVA** | | **AVA** | | **AVA** | |
|  | **FE** | **RE** | **FE** | **RE** | **FE** | **RE** | **FE** | **RE** | **FE** | **RE** |
| **FC** |  |  |  |  |  |  |  |  | -3.74e-06 | -4.10e-06 |
|  |  |  |  |  |  |  |  |  | (6.76e-06) | (5.91e-06) |
| **EA** | 0.2426*** | 0.2423*** | 0.2423*** | 0.2421*** | 0.2437*** | 0.2426*** | 0.2353*** | 0.2340*** | 0.2353*** | 0.2341*** |
|  | (0.0595) | (0.0527) | (0.0596) | (0.0530) | (0.0596) | (0.0532) | (0.0676) | (0.06066) | (0.0676) | (0.0609) |
| **ARME** |  |  |  |  |  |  |  |  |  |  |
|  |  |  |  |  |  |  |  |  |  |  |
| **ARMI** |  |  |  |  |  |  | 0.2588 | 0.2717 | 0.2583 | 0.2707 |
|  |  |  |  |  |  |  | (0.1920) | (0.1890) | (0.1923) | (0.1894) |
| **FDI** |  |  | 0.00105*** | 0.00102*** | 0.00106*** | 0.00103*** | 0.00092*** | 0.00090*** | 0.00092*** | 0.000904*** |
|  |  |  | (0.00032) | (0.00033) | (0.00033) | (0.00034) | (0.00030) | (0.00031) | (0.00030) | (0.000314) |
| **ER** |  |  |  |  | 0.00293*** | 0.00261*** | 0.0027*** | 0.00249*** | 0.00273*** | 0.00250*** |
|  |  |  |  |  | (0.000601) | (0.000686) | (0.00072) | (0.00070) | (0.00073) | (0.0007053) |
| **Constant** | 1.0520 | 1.0537 | 1.0454 | 1.04691 | 0.9295 | 0.9470 | 0.6891 | 0.6905 | 0.6905 | 0.6931 |
| **No of Countries** | 32 | 32 | 32 | 32 | 32 | 32 | 32 | 32 | 32 | 32 |
| **No of years** | 22 | 22 | 22 | 22 | 22 | 22 | 22 | 22 | 22 | 22 |
| **R^2^ Within** | 0.3671 | 0.3671 | 0.3681 | 0.3680 | 0.3724 | 0.3724 | 0.3829 | 0.3828 | 0.3829 | 0.3829 |
| **R^2^ Between** | 0.5439 | 0.5439 | 0.5431 | 0.5431 | 0.5527 | 0.5540 | 0.5769 | 0.5786 | 0.5770 | 0.5787 |
| **R^2^ Overall** | 0.5180 | 0.5180 | 0.5175 | 0.5175 | 0.5261 | 0.5273 | 0.5484 | 0.5499 | 0.5486 | 0.5500 |

**S3.2 Appendix. Panel regression for high income countries**

Note: The symbols *, **, and *** represents 10%, 5%, and 1% significance level, respectively. Parentheses represent the robust standard error. FE and RE represent the Fixed effect and Random effect, respectively.

| **Low-Income Level** | | | | | | | | | | | |
| --- | --- | --- | --- | --- | --- | --- | --- | --- | --- | --- | --- |
| **Equation** | **Y = f (FC)** | | **Y = f (FC EA)** | | **Y = f (FC EA ER)** | | **Y = f (FC EA ER ARMI1** | | **Y = f (FC EA ER ARMI FDI)** | | |
| **Variables** | **AVA** | | **AVA** | | **AVA** | | **AVA** | | **AVA** | | |
|  | **FE** | **RE** | **FE** | **RE** | **FE** | **RE** | **FE** | **RE** | **FE** | **RE** |  |
| **FC** | -0.1815*** | -0.1892*** | -0.0919 | 0.0733 | -0.0756 | -0.0912 | -0.0736 | -0.08745 | -0.0696 | -0.0944 |  |
|  | (0.0572) | (0.0586) | (0.0733) | (0.0696) | (0.0701) | (0.0700) | (0.0696) | (0.0697) | (0.0697) | (0.0703) |  |
| **EA** |  |  | 0.1719* | 0.1619** | 0.1646* | 0.1591** | 0.1605* | 0.1560** | 0.1658* | 0.1560** |  |
|  |  |  | (0.0850) | (0.0695) | (0.0778) | (0.0667) | (0.0773) | (0.0675) | (0.0799) | (0.0629) |  |
| **ARME** |  |  |  |  |  |  |  |  |  |  |  |
|  |  |  |  |  |  |  |  |  |  |  |  |
| **ARMI** |  |  |  |  |  |  | 0.2235 | 0.2178 | 0.2339 | 0.2217 |  |
|  |  |  |  |  |  |  | (0.1673) | (0.1661) | (0.1664) | (0.1646) |  |
| **FDI** |  |  |  |  |  |  |  |  | 0.0581** | 0.0419 |  |
|  |  |  |  |  |  |  |  |  | (0.0734) | (0.0713) |  |
| **ER** |  |  |  |  | -0.0024** | -0.0021** | -0.0025* | -0.0022** | -0.0024** | -0.0020** |  |
|  |  |  |  |  | (0.00103) | (0.000886) | (0.00106) | (0.00092) | (0.00106) | (0.0008) |  |
| **Constant** | 29.1275 | 29.2101 | 17.1164 | 17.9253 | 19.4165 | 19.7143 | 19.4304 | 19.6745 | 18.7692 | 19.4104 |  |
| **No of Countries** | 11 | 11 | 11 | 11 | 11 | 11 | 11 | 11 | 11 | 11 |  |
| **No of years** | 22 | 22 | 22 | 22 | 22 | 22 | 22 | 22 | 22 | 22 |  |
| **R^2^ Within** | 0.1469 | 0.1469 | 0.2304 | 0.2298 | 0.2760 | 0.2749 | 0.2809 | 0.2799 | 0.2847 | 0.2817 |  |
| **R^2^ Between** | 0.4389 | 0.4389 | 0.2861 | 0.3242 | 0.1342 | 0.1913 | 0.1284 | 0.1784 | 0.1037 | 0.1888 |  |
| **R^2^ Overall** | 0.3341 | 0.3341 | 0.2705 | 0.2961 | 0.1673 | 0.2082 | 0.1640 | 0.2006 | 0.1448 | 0.2087 |  |

**S3.3 Appendix. Panel regression for low-income countries**

Note: The symbols *, **, and *** represents 10%, 5%, and 1% significance level, respectively.

Parentheses represent the robust standard error. FE and RE represent the Fixed effect and Random effect, respectively.

**S3.4 Appendix. Panel regression for lower middle-income countries**

| **Lower-Middle Income Level** | | | | | | | | | | |
| --- | --- | --- | --- | --- | --- | --- | --- | --- | --- | --- |
| **Equation** | **Y= f (EA)** | | **Y= f (EA FDI)** | | **Y= f (EA FDI FC)** | | **Y= f (EA FDI FC ARME)** | | **Y= f (EA FDI FC ARME ER)** | |
| **Variables** | **AVA** | | **AVA** | | **AVA** | | **AVA** | | **AVA** | |
|  | **FE** | **RE** | **FE** | **RE** | **FE** | **RE** | **FE** | **RE** | **FE** | **RE** |
| **FC** |  |  |  |  | 0.00064 | -0.0010 | 0.00043 | -0.0011 | 0.000841 | -0.0011 |
|  |  |  |  |  | (0.00603) | (0.0046) | (0.0059) | (0.0045) | (0.0058) | (0.0046) |
| **EA** | 0.2751*** | 0.2751*** | 0.2589*** | 0.2685*** | 0.2599*** | 0.2671*** | 0.2560*** | 0.2622*** | 0.2692*** | 0.2722*** |
|  | (0.0164) | (0.0715) | (0.0760) | (0.0704) | (0.0801) | (0.0728) | (0.0761) | (0.0688) | (0.0768) | (0.0696) |
| **ARME** |  |  |  |  |  |  | 0.0839 | 0.0942 | 0.0829 | 0.0934 |
|  |  |  |  |  |  |  | (0.0852) | (0.0667) | (0.0842) | (0.0668) |
| **ARMI** |  |  |  |  |  |  |  |  |  |  |
|  |  |  |  |  |  |  |  |  |  |  |
| **FDI** |  |  | -0.1663* | -0.1536* | -0.1661* | -0.1533* | -0.1610* | -0.1452* | -0.1536* | -0.1402* |
|  |  |  | (0.0836) | (0.0867) | (0.0840) | (-0.1533) | (0.0807) | (0.0831) | (0.0824) | (0.0842) |
| **ER** |  |  |  |  |  |  |  |  | 0.00016*** | 0.00015*** |
|  |  |  |  |  |  |  |  |  | (0.0000464) | (0.0000446) |
| **Constant** | 6.1247 | 5.8096 | 6.8880 | 6.5029 | 6.7841 | 6.6559 | 6.5107 | 6.3408 | 5.6348 | 5.6582 |
| **No of Countries** | 28 | 28 | 28 | 28 | 28 | 28 | 28 | 28 | 28 | 28 |
| **No of years** | 22 | 22 | 22 | 22 | 022 | 22 | 22 | 22 | 22 | 22 |
| **R^2^ Within** | 0.2941 | 0.2941 | 0.3071 | 0.3070 | 0.3072 | 0.3067 | 0.3147 | 0.3142 | 0.3363 | 0.3355 |
| **R^2^ Between** | 0.6099 | 0.6099 | 0.5844 | 0.5878 | 0.5792 | 0.5951 | 0.6111 | 0.6240 | 0.5619 | 0.5875 |
| **R^2^ Overall** | 0.5508 | 0.5508 | 0.5313 | 0.5342 | 0.5272 | 0.5400 | 0.5574 | 0.5683 | 0.5229 | 0.5438 |

Note: The symbols *, **, and *** represents 10%, 5%, and 1% significance level, respectively.

Parentheses represent the robust standard error. FE and RE represent the Fixed effect and Random effect, respectively.

| **Upper-Middle Income** | | | | | | | | | | | | | |
| --- | --- | --- | --- | --- | --- | --- | --- | --- | --- | --- | --- | --- | --- |
| **Equation** | **Y= f (EA)** | | **Y = f (EA ARME)** | | **Y = f (EA ARME ARMI)** | | **Y = f (EA ARME ARMI ER)** | | **Y = f (EA ARME ARMI ER FDI)** | | **Y = f (EA ARME ARMI ER FDI FC)** | |  |
| **Variables** | **AVA** | | **AVA** | | **AVA** | | **AVA** | | **AVA** | | **AVA** | |  |
|  | **FE** | **RE** | **FE** | **RE** | **FE** | **RE** | **FE** | **RE** | **FE** | **RE** | **FE** | **RE** |  |
| **FC** |  |  |  |  |  |  |  |  |  |  | 0.00016 | 0.00041 |  |
|  |  |  |  |  |  |  |  |  |  |  | (0.00108) | (0.00093) |  |
| **EA** | 0.2855*** | 0.2646*** | 0.2552*** | 0.2390*** | 0.2503*** | 0.2338*** | 0.2527*** | 0.2351*** | 0.2514*** | 0.2340*** | 0.2523*** | 0.2359*** |  |
|  | (0.0489) | (0.0405) | (0.0418) | (0.0358) | (0.0435) | (0.0359) | (0.0438) | (0.0356) | (0.0462) | (0.0382) | (0.0469) | (0.0382) |  |
| **ARME** |  |  | 0.4860*** | 0.4799*** | 0.4738** | 0.4680*** | 0.4712** | 0.4627*** | 0.4681** | 0.4600*** | 0.4690** | 0.4611*** |  |
|  |  |  | (0.1942) | (0.1785) | (0.2041) | (0.1812) | (0.2007) | (0.1784) | (0.1987) | (0.1762) | (0.2002) | (0.1759) |  |
| **ARMI** |  |  |  |  | 0.1735 | 0.2243 | 0.1672 | 0.2281 | 0.1786 | 0.2396 | 0.1742 | 0.2279 |  |
|  |  |  |  |  | (0.3450) | (0.3052) | (0.3436) | (0.3044) | (0.3463) | (0.3057) | (0.3510) | (0.3093) |  |
| **FDI** |  |  |  |  |  |  |  |  | 0.0079 | 0.0094 | 0.0078 | 0.00942 |  |
|  |  |  |  |  |  |  |  |  | (0.0533) | (0.0516) | (0.0533) | (0.0517) |  |
| **ER** |  |  |  |  |  |  | 0.00030 | 0.00030*** | 0.00030 | 0.00030*** | 0.00031 | 0.00031*** |  |
|  |  |  |  |  |  |  | (0.00024) | (0.0000813) | (0.00024) | (0.0000827) | (0.00024) | (0.0000804) |  |
| **Constant** | 1.7303 | 2.1585 | 1.3183 | 1.6636 | 1.2059 | 1.4867 | 1.0794 | 1.3746 | 1.0639 | 1.3454 | 1.0143 | 1.2325 |  |
| **No. Of Countries** | 30 | 30 | 30 | 30 | 30 | 30 | 30 | 30 | 30 | 30 | 30 | 30 |  |
| **No. of Years** | 22 | 22 | 22 | 22 | 22 | 22 | 22 | 22 | 22 | 22 | 22 | 22 |  |
| **R^2^ Within** | 0.3573 | 0.3573 | 0.3952 | 0.3951 | 0.3963 | 0.3961 | 0.3973 | 0.3970 | 0.3976 | 0.3973 | 0.3976 | 0.3972 |  |
| **R^2^ Between** | 0.4884 | 0.4884 | 0.5226 | 0.5223 | 0.5239 | 0.5238 | 0.5428 | 0.5438 | 0.5420 | 0.5429 | 0.5462 | 0.5529 |  |
| **R^2^ Overall** | 0.4287 | 0.4287 | 0.4667 | 0.4666 | 0.4689 | 0.4694 | 0.4817 | 0.4830 | 0.4814 | 0.4828 | 0.4843 | 0.4896 |  |

**S3.5 Appendix. Panel regression for lower upper middle-income countries**

Note: The symbols *, **, and *** represents 10%, 5%, and 1% significance level, respectively.

Parentheses represent the robust standard error. FE and RE represent the Fixed effect and Random effect, respectively.
