# Supplementary material for: Impact of economic globalisation on value-added agriculture, globally
Source: PLoS One. 2023 Jul 21;18(7):e0289128. doi: 10.1371/journal.pone.0289128 (PMC10361532; doi:10.1371/journal.pone.0289128)
Supplement: S4 Appendix — (DOCX) [file pone.0289128.s004.docx]

**S4 Appendix.** **Fixed effect and Random effect estimates for the final stepwise model**

| **Income Levels** | | | | | | | | | | | |
| --- | --- | --- | --- | --- | --- | --- | --- | --- | --- | --- | --- |
|  | **All Countries** | | **High-Income** | | **Low-Income** | | **Lower-Middle** | | **Upper- Middle** | | |
| **Equation** | **Y=f (EA ARMI FC FDI ER)** | | **Y=f (EA FDI ER ARMI FC)** | | **Y=f (FC EA ER ARMI FDI)** | | **Y=f (EA FDI FC ARME ER)** | | **Y=f (EA ARME ARMI ER FDI FC)** | | |
| **Variables** | **AVA** | | **AVA** | | **AVA** | | **AVA** | | **AVA** | | |
|  | **FE** | **RE** | **FE** | **RE** | **FE** | **RE** | **FE** | **RE** | **FE** | **RE** |  |
| **FC** | -6.79e-06 | -0.0000133 | -3.74e-06 | -4.10e-06 | -0.0696296 | -0.0944 | 0.000841 | -0.00117 | 0.000165 | 0.000418 |  |
|  | (0.0000104) | (0.0000126) | (6.76e-06) | (5.91e-06) | (0.0697) | (0.0703) | (0.0058) | (0.0046) | (0.00108) | (0.00093) |  |
| **EA** | 0.2616*** | 0.2825*** | 0.2353*** | 0.2341*** | 0.1658* | 0.1560** | 0.2692*** | 0.2722*** | 0.2523*** | 0.2359*** |  |
|  | (0.0476) | (0.0400) | (0.0676) | (0.0609) | (0.0799) | (0.0629) | (0.0768) | (0.0696) | (0.0469) | (0.0382) |  |
| **ARME** |  |  |  |  |  |  | 0.0829 | 0.0934 | 0.4690** | 0.4611*** |  |
|  |  |  |  |  |  |  | (0.0842) | (0.0668) | (0.2002) | (0.1759) |  |
| **ARMI** | 0.0029 | -0.0098 | 0.2583 | 0.2707 | 0.2339 | 0.2217 |  |  | 0.1742 | 0.2279 |  |
|  | (0.1416) | (0.1344) | (0.1923) | (0.1894) | (0.1664) | (0.1646) |  |  | (0.3510) | (0.3093) |  |
| **FDI** | 0.0012 | 0.0009428 | 0.000923*** | 0.000904*** | 0.0581** | 0.0419 | -0.1536* | -0.1402* | 0.0078 | 0.0094 |  |
|  | (0.0019) | (0.0019) | (0.00030) | (0.000314) | (0.0734) | (0.0713) | (0.0824) | (0.0842) | (0.0533) | (0.0517) |  |
| **ER** | 0.000143*** | 0.00015*** | 0.0027*** | 0.00250*** | -0.0024** | -0.0020** | 0.000161*** | 0.000150*** | 0.00031 | 0.00031*** |  |
|  | (0.0000544) | (0.0000471) | (0.00073) | (0.0007053) | (0.0010) | (0.00085) | (0.0000464) | (0.000044) | (0.00024) | (0.0000804) |  |
| **Constant** | 3.7150 | 3.2085 | 0.6905 | 0.6931 | 18.7692 | 19.4104 | 5.6348 | 5.6582 | 1.0143 | 1.2325 |  |
| **No of Countries** | 101 | 101 | 32 | 32 | 11 | 11 | 28 | 28 | 30 | 30 |  |
| **No of years** | 22 | 22 | 22 | 22 | 22 | 22 | 22 | 22 | 22 | 22 |  |
| **R^2^ Within** | 0.2865 | 0.2864 | 0.3829 | 0.3829 | 0.2847 | 0.2817 | 0.3363 | 0.3355 | 0.3976 | 0.3972 |  |
| **R^2^ Between** | 0.7988 | 0.7990 | 0.5770 | 0.5787 | 0.1037 | 0.1888 | 0.5619 | 0.5875 | 0.5462 | 0.5529 |  |
| **R^2^ Overall** | 0.7643 | 0.7645 | 0.5486 | 0.5500 | 0.1448 | 0.2087 | 0.5229 | 0.5438 | 0.4843 | 0.4896 |  |

Note: The symbols *, **, and *** represents 10%, 5%, and 1% significance level, respectively. Parentheses represent the robust standard error. FE and RE represent the Fixed effect and Random effect, respectively
