# Supplementary material for: Impact of economic globalisation on value-added agriculture, globally
Source: PLoS One. 2023 Jul 21;18(7):e0289128. doi: 10.1371/journal.pone.0289128 (PMC10361532; doi:10.1371/journal.pone.0289128)
Supplement: Attachment — Submitted filename: Review report PONE 23-05826.docx [file pone.0289128.s006.docx]

Review report PONE-D-23-05826 Impact of Economic Globalisation on Value-Added Agriculture, Globally

**Abstract**

The topic of this article is Impact of Economic Globalisation on Value-Added Agriculture. However, the abstract does not contain any information about economic globalisation.

“The panel data regression with the stepwise method was employed to quantify the impact of economic globalisation in 101 countries between 2000 to 2021.” Quantify the impact of economic globalisation on? Grammatical error.

**Introduction**

“In addition, Globalisation can transform rural agriculture into more commercialised and value-based agriculture and improve the rural community’s living conditions [5].” Why Globalisation is a capital letter?

Are these variables refer to economic globalisation? “Trade, Exchange Rate (ER) and Foreign Direct Investment (FDI) are crucial in the global agricultural sector.” If yes, please write it and inform readers.

The in-text citation is not consistent throughout the article. Example, “Nyiwul and Koirala [7].”, “Manamba Epaphra (2017)”, “Schuh [10]”

This study is about global. Why specifically mentioned about China? “In China, according to a performance index, agriculture is receiving more foreign FDI, but not at a rate that is satisfactory given the size of the industry [8, 9].”?

Why did you mention this? “Further findings demonstrate that Employment in Agriculture (EA) and Fertilizer Consumption (FC) affect AVA.”

Any intention to include this in the article? “Agriculture Raw Material Exports (ARME) and Agriculture Raw Material Imports (ARMI) effects the foreign currency inflows and outflows of nations.”

This section is poorly written. The authors introduced all the keywords, then proceed to write the purpose of the study. This research is not supported by strong research problem.

**Literature Review**

Since the authors did not guide the readers to understand the research problem well from the beginning, the contents of this section are also unable to add value to the readers.

It is very obvious that the authors only summarise the past literature on the variables that the authors intend to study. The authors were unable to provide a critical evaluation of these works.

Why break the section into High-income, Low-Income Level, Lower Middle-Income Level and Upper Middle-Income Level?

This section is poorly written. The authors failed to integrate arguments into the review. Authors simply summarising their readings.

**Data and Methodology**

This section is weakly written. The authors only present the variables intended to study. There is no theoretical framework or hypothesis to support the methods. All the variables and methods are basically formed by the readers without any literature support.

**Results**

This manuscript only stated the findings without interpretation.

**Discussion**

This section is poorly written. Too much description and not enough analysis. The authors basically cited a lot of literature to end a sentence.

**Policy Implications**

This section is incompetently written. The authors basically mentioned “the government should …” for all the variables examined in this study.

**Conclusion**

The authors only summarise the study.

The conclusion is intended to help the reader understand why your research should matter to them after they have finished reading the paper. A conclusion is not merely a summary of your points or a re-statement of your research problem but a synthesis of key points.

**Overall**

It is a poorly written article. It seems like an assignment for undergraduate students. It does not meet the expectation to be published in a high-impact journal.
