# Supplementary material for: Impact of economic globalisation on value-added agriculture, globally
Source: PLoS One. 2023 Jul 21;18(7):e0289128. doi: 10.1371/journal.pone.0289128 (PMC10361532; doi:10.1371/journal.pone.0289128)
Supplement: Attachment — Submitted filename: Response to Reviewers.docx [file pone.0289128.s007.docx]

**Point by point response to editor and reviewers**

Dear Editor and Reviewers,

We would like to express our profound appreciation to the editor and the reviewers for the valuable comments and suggestions made on our manuscript which were very helpful in revising and improving it.

Please note that the line numbers referred in this document is aligned with the revised manuscript which has track changes.

| **Comments of the Reviewer** | **Comments of the Authors** |
| --- | --- |
| 1. Is the manuscript technically sound, and do the data support the conclusions?  The manuscript must describe a technically sound piece of scientific research with data that supports the conclusions. Experiments must have been conducted rigorously, with appropriate controls, replication, and sample sizes. The conclusions must be drawn appropriately based on the data presented.  **Reviewer #1: Partly**  **Reviewer #2: Yes**  **Reviewer #3: No**  **Reviewer #4: No**  **Reviewer #5: Partly** | Well noted. Thank you for your comment. Comments have been incorporated in the revised version |
| **2. Has the statistical analysis been performed appropriately and rigorously?**  **Reviewer #1: No**  **Reviewer #2: Yes**  **Reviewer #3: No**  **Reviewer #4: Yes**  **Reviewer #5: No** | Duly noted! Thank you! Response well considered and expanded the statistical analysis. |
| 3. Have the authors made all data underlying the findings in their manuscript fully available?  The [PLOS Data policy](http://www.plosone.org/static/policies.action#sharing) requires authors to make all data underlying the findings described in their manuscript fully available without restriction, with rare exception (please refer to the Data Availability Statement in the manuscript PDF file). The data should be provided as part of the manuscript or its supporting information or deposited to a public repository. For example, in addition to summary statistics, the data points behind means, medians and variance measures should be available. If there are restrictions on publicly sharing data—e.g. participant privacy or use of data from a third party—those must be specified.  **Reviewer #1: Yes**  **Reviewer #2: Yes**  **Reviewer #3: No**  **Reviewer #4: Yes**  **Reviewer #5: Yes** | Noted with thanks! authors ensured all data underlying the findings are available in the manuscript. |
| 4. Is the manuscript presented in an intelligible fashion and written in standard English?  PLOS ONE does not copyedit accepted manuscripts, so the language in submitted articles must be clear, correct, and unambiguous. Any typographical or grammatical errors should be corrected at revision, so please note any specific errors here.  **Reviewer #1: No**  **Reviewer #2: Yes**  **Reviewer #3: No**  **Reviewer #4: No**  **Reviewer #5: Yes** | Comment well noted with thanks. Language has been improved with the assistance of professional language editor. |
| **REVIEWER #1** | |
| **Comment 1** |  |
| The manuscript does not have a strong theoretical foundation. I recommend the author add international trade theory and how the research variable represents the theory. | Thanks for this. Having taken due note of your feedback, we have now included two well-established trade theories, the Heckscher-Ohlin theory and the New Trade theory. We appreciate your valuable feedback which now strengthens the theoretical foundation of the research article. We have also explained how the research variables represent these theories. This update can be found in the revised manuscript on lines 72 to 82.  “…There are several interrelated trade theories that impact AVA. Two of the most influential theories are the Heckscher-Ohlin theory and the New Trade theory. The Heckscher-Ohlin theory suggests that countries will specialize in producing goods that use their abundant factors of production, while the New Trade theory suggests that economies of scale and increasing returns to specialisation can create a comparative advantage and lead to trade. Both theories can lead to increased value addition in agriculture as countries and firms specialise in producing goods where they have a comparative advantage and invest in research and development to improve the quality of their products. In summary, international trade can increase value addition in agriculture…” |
| **Comment 2** |  |
| The author uses 101 countries and categorized them based on GDP per capita, but the authors need to mention the source of the categorization: What is the World Bank, IMF or other sources? | Thanks for the comment. Authors have used four income- level categorizations of countries by the World Bank based on their Gross National Income (GNI) per capita.   1. Low-income countries: with a GNI per capita of $1,045 or less. 2. Lower-middle-income countries: with a GNI per capita between $1,046 and $4,125. 3. Upper-middle-income countries: with a GNI per capita between $4,126 and $12,735. 4. High-income countries: with a GNI per capita of $12,736 or more.   Comment has been incorporated in the revised manuscript and base categorization mentioned from lines 406 to 411. |
| **Comment 3** |  |
| Why do the authors make different models for each country category? This makes the conclusion and policy implications inappropriate. Moreover, the assumption of economic globalization is all countries face the same external conditions, so the author needs to make the same model between countries. Literature review and incomplete data cannot be the justification for differences in models between countries. | Thanks for the valuable comment made. Authors have used all variables in the initial model at each income level. However, to finalise the final model of each income level, the study utilised the stepwise method. This enables managing large amount of potential predictor variables, fine- tuning the model to choose the best predictor variables from the available options in each income category. The Stepwise technique can simplify the model, reduce overfitting, improve prediction accuracy and avoid irrelevant or redundant variables that do not contribute to the explanation of the Agriculture Value Added. Hence, the authors believed that best final models generated by the stepwise technique would be most appropriate to compare each income category. |
| **Comment 4** |  |
| The author is expected to check again Table 3. For example, is it right to choose RE for all countries model? Even though the F-Test and Hausman Test show H_0_ is rejected so the best model is Fixed Effect. | Thanks very much. This was corrected in revised manuscript in line 651 to 655.  “In conclusion, Table 3 indicates that the FE model is not valid for -income and upper-middle-income levels. In contrast, the FE model best fit for all countries and low-income groups. These findings emphasise the importance of considering the specific income level when selecting the appropriate panel data model”. |
| **Comment 5** |  |
| The author should also choose only 1 best model (RE or FE) to be displayed in Table 4. In addition, what is meant by numbers in brackets in Table 4? probability value or standard error? If it is the probability: several variables should be significant (p-value is smaller than 0.05). | Well noted. Table 4 was moved to appendix S4 and only 1 best model (RE or FE) for global level and income levels are shown in Table 4 in revised manuscript. Responding to your query, the numbers within the brackets “()” in Table 4 represent the robust standard errors and it is mentioned under the table. |
| **Comment 6** |  |
| Please the authors to improve discussions, policy implications, and conclusion sections based on the revision of the results section | Thanks for the valuable feedback. We have thoroughly revised the discussion, policy implications, and conclusion sections, based on the revised results section from line number 681 to 930. We consider that our revised sections provide a more insightful and comprehensive understanding of the significance of our study. |
| **Comment 7** |  |
| The author needs to add limitations and further research in the conclusion section. | Thank you for the feedback. Do refer to lines 843 to 835 for a detailed discussion on the limitations of the study and suggestions for future research. We believe these additions will enhance the overall contribution to our research. |
| **Comment 8** |  |
| Writing references must be fixed to adjust the PLOS ONE journal template | We have made the changes accordingly as per the PLOS One journal template. |
| **Comment 9** |  |
| The author should use a professional language editor to improve the quality of this manuscript. | Noted with thanks. The paper has been revised thoroughly and in-depth copy edit has conducted by an experienced copy-editor. We trust that the revised manuscript is free from any language errors. |
| **REVIEWER #2** | |
| **Comment 1** |  |
| Title of the study should be changed, for instance, "The Role of Economic Globalization in the Transformation of Agricultural Value Chains" | Thanks very much for the suggested title. The purpose of current study is to investigate whether economic globalisation has had an impact on agriculture value added globally. Correspondingly, the study has less focus on agriculture value chains. Hence, the authors believed that current title is most appropriate. |
| **Comment 2** |  |
| Introduction: Add possible research questions and linked them with the study's objectives. | Thank you for your comment. We have taken cognisance and have included possible research questions that are linked to the study's objectives. Do refer to the revised manuscript from lines 162 to 174. We trust that such additions have addressed your concern and improved the overall quality of the research. |
| **Comment 3** |  |
| Add latest literature up to 2023, for instance, - Gyamfi, B. A., Onifade, S. T., Erdoğan, S., & Ali, E. B. (2023). Colligating ecological footprint and economic globalization after COP21: Insights from agricultural value-added and natural resources rents in the E7 economies. International Journal of Sustainable Development & World Ecology, 1-15. - Zaman, K. (2023). The Future of Financial Support for Developing Countries: Regional and Islamic Monetary Funds. Politica, 1(1), 1–8. <https://doi.org/10.5281/zenodo.7610145> - Raihan, A. (2023). An econometric evaluation of the effects of economic growth, energy use, and agricultural value added on carbon dioxide emissions in Vietnam. Asia-Pacific Journal of Regional Science, 1-32. - Khan, M. (2023). Shifting Gender Roles in Society and the Workplace: Implications for Environmental Sustainability. Politica, 1(1), 9–25. <https://doi.org/10.5281/zenodo.7634130> - Chen, Y., & Zhang, Y. (2023). Services Development, Technological Innovation, and the Embedded Location of the Agricultural Global Value Chain. Sustainability, 15(3), 2673. - Fatima, S. (2023). Rural Development and Education: Critical Strategies for Ending Child Marriages. Archives of the Social Sciences: A Journal of Collaborative Memory , 1(1), 1-15. <https://doi.org/10.5281/zenodo.7556588> - Xu, Y., Li, C., & Wang, J. How does agricultural global value chain affect ecological footprint? The moderating role of environmental regulation. Sustainable Development. - Aqib, M., & Zaman, K. (2023). Greening the Workforce: The Power of Investing in Human Capital. Archives of the Social Sciences: A Journal of Collaborative Memory, 1(1), 31–51. <https://doi.org/10.5281/zenodo.7620041> - Raihan, A., Muhtasim, D. A., Farhana, S., Hasan, M. A. U., Pavel, M. I., Faruk, O., ... & Mahmood, A. (2023). An econometric analysis of Greenhouse gas emissions from different agricultural factors in Bangladesh. Energy Nexus, 100179. - Khan, M, T., & Imran, M. (2023). Unveiling the Carbon Footprint of Europe and Central Asia: Insights into the Impact of Key Factors on CO2 Emissions. Archives of the Social Sciences: A Journal of Collaborative Memory, 1(1), 52–66. <https://doi.org/10.5281/zenodo.7669782> - Yan, B., Xia, Y., & Jiang, X. (2023). Carbon Productivity and Value-Added Generations: Regional Heterogeneity along Global Value Chain?. Structural Change and Economic Dynamics. | Thank you for suggesting these papers. We have duly incorporated several of them into our Introduction and Literature Review sections.  “…Another study explores the relationship between economic globalization, agricultural value-added, and ecological footprint in the E7 nations which are Brazil, China, India, Indonesia, Mexico, Russia and Turkey. The results indicate that these factors have contributed to environmental deterioration, and policymakers should implement environmental damage cost and maintain strategic resource control measures for a sustainable environment”. <https://doi.org/10.1080/13504509.2023.2166141>  This was corrected in revised manuscript in line 206 to 211.  “…. Another study by Raihan (2023) suggested that Vietnam should enhance AVA by introducing modern agro-based technology and promoting sustainable agriculture, such as low-carbon agriculture systems and the use of renewable energy, and by avoiding excessive use of fertilizers and pesticides.”. <https://doi.org/10.5281/zenodo.7634130>  This was corrected in revised manuscript in line 349 to 352.  “…Agriculture is vital for a country's economy, and enhancing the embedded location of agricultural value chains is crucial for its modernization. The global division of labor has led to an increase in the role of the agricultural global value chain, shifting agricultural trade from single-country production to multi-country production. This has refined the international division of labor in agriculture and extended production chains, leading to national agriculture being included in the global division of labor system dominated by multinational corporations.” <https://doi.org/10.5281/zenodo.7556588>  This was corrected in revised manuscript in line 113 to 120.  “…Another study by Raihan [57] suggested that Vietnam should enhance AVA by introducing modern agro-based technology and promoting sustainable agriculture, such as low-carbon agriculture systems and the use of renewable energy, and by avoiding excessive use of fertilizers and pesticides.”  <https://doi.org/10.5281/zenodo.7620041>  This was corrected in revised manuscript in line 349 to 352.  “Prior investigations found a positive association between FC and GHG emissions, indicating a need to avoid excessive use of fertilizers and pesticides in sustainable agriculture. The government should impose restrictions on the use of chemical fertilizers and engage in research and development to develop environmentally sustainable fertilizers and new crops that do not rely on hazardous fertilizers. Organic and low-carbon agriculture systems should be encouraged to reduce emissions and improve carbon sequestration. The study suggests policymakers promote organic farming, tunnel farming, no-till farming, and limit fertilizer use to reduce environmental impact.” <https://doi.org/10.1016/j.nexus.2023.100179>  This was corrected in revised manuscript in line 120 to 127. |
| **Comment 4** |  |
| Literature review: Add missing gaps and contribution of the study. | Your comment is welcome, and thus we have strengthened the contribution of the study and literature gap in the revised manuscript from lines 142 to 187. |
| **Comment 5** |  |
| Add possible research hypotheses in the literature review. | Thank you for your suggestion to include possible research hypotheses in our literature review. Our study investigates the impact of economic globalization on agriculture value added at four income levels (low, lower middle, upper middle, and high), as well as at a global level. Accordingly, we have tested the significance of seven independent variables, resulting in a total of 35 hypotheses. While we appreciate the importance of including such hypotheses in a research paper, we were concerned that listing all 35 could disrupt the flow of the literature review. We have provided clear research objectives and questions and believe that our readers will understand the implied hypotheses. This has been amended in line 392 to 397. |
| **Comment 6** |  |
| Compare and contrast the used statistical panel technique with the cross-panel techniques, for instance, - Guschanski, A., & Onaran, Ö. (2023). Global Value Chain Participation and the Labour Share: Industry‐level Evidence from Emerging Economies. Development and Change, 54(1), 31-63. - Zaman, K. (2023). A Note on Cross-Panel Data Techniques. Latest Developments in Econometrics, 1(1), 1-7. <https://doi.org/10.5281/zenodo.7565625> - Zhang, Y., Liao, C., & Pan, B. (2023). Ecological unequal exchange between China and European Union: An investigation from global value chains and carbon emissions viewpoint. Atmospheric Pollution Research, 101661. | Thank you for your feedback. We have revised our manuscript by including a comparison of the statistical panel technique with cross-panel techniques and citing relevant articles to support our discussion.  “Panel data regression combines cross-sectional and time-series data to analyse changes in variables within and between entities over time. Cross-panel analysis is a type of panel data analysis that compares variable interdependence across different nations, allowing researchers to evaluate policy effects and identify relationships between variables across various periods and cross-sections. Cross-panel regression provides valuable insights into the global impact of policies by exploring the simultaneous movement of both dependent and independent variables. The utilization of the cross-panel technique is also viable for conducting research on this area” <https://doi.org/10.5281/zenodo.7565625>  This was added in revised manuscript in line 384 to 391. |
| **Comment 7** |  |
| Add possible research limitations and future directions at the end. | Duly Noted, we have added limitations and further research in before the conclusion section. You can refer from line 843 to 877. |
| **REVIEWER #3** | |
| **Comment 1** |  |
| Abstract The topic of this article is Impact of Economic Globalisation on Value-Added Agriculture. However, the abstract does not contain any information about economic globalisation. “The panel data regression with the stepwise method was employed to quantify the impact of economic globalisation in 101 countries between 2000 to 2021.” Quantify the impact of economic globalisation on? Grammatical error. | Thank you for highlighting this. We regret the oversight in the abstract and accordingly have made the necessary revisions to clearly underline the meaning of economic globalization. Do refer to the updated abstract for further details in lines 28 to 30. Additionally, we have corrected the grammatical error in the sentence you have mentioned, refer line to 33 to 37. |
| **Comment 2** |  |
| Introduction “In addition, Globalisation can transform rural agriculture into more commercialised and value-based agriculture and improve the rural community’s living conditions [5].” Why Globalisation is a capital letter?  Are these variables refer to economic globalisation? “Trade, Exchange Rate (ER) and Foreign Direct Investment (FDI) are crucial in the global agricultural sector.” If yes, please write it and inform readers.  The in-text citation is not consistent throughout the article. Example, “Nyiwul and Koirala [7].”, “Manamba Epaphra (2017)”, “Schuh [10]”  This study is about global. Why specifically mentioned about China? “In China, according to a performance index, agriculture is receiving more foreign FDI, but not at a rate that is satisfactory given the size of the industry [8, 9].”? Why did you mention this? “Further findings demonstrate that Employment in Agriculture (EA) and Fertilizer Consumption (FC) affect AVA.” Any intention to include this in the article?  “Agriculture Raw Material Exports (ARME) and Agriculture Raw Material Imports (ARMI) effects the foreign currency inflows and outflows of nations.” This section is poorly written. The authors introduced all the keywords, then proceed to write the purpose of the study. This research is not supported by strong research problem. | Thank you for pointing the mistake. We have corrected the capital letter. Please refer the revised manuscript in line 58.  According to reference of “Nugroho, A. D., Bhagat, P. R., Magda, R., & Lakner, Z. 2021. The impacts of economic globalization on agricultural value added in developing countries. PLOS ONE [Online], 16(11), pp.e0260043. Available at: <https://doi.org/10.1371/journal.pone.0260043>” Exchange Rate (ER) and Foreign Direct Investment (FDI) were the few key factor taken for economic globalization. Furthermore, the article recommend that further research can be carried out by many trade economists.  The in-text citation had been corrected.  Thank you for your feedback. We appreciate your comment on the clarity of the section related to Agriculture Raw Material Exports (ARME) and Agriculture Raw Material Imports (ARMI). We have revised the section to provide a clearer explanation of the variables and how they relate to the research problem. We have also improved the introduction to better articulate the research problem and the purpose of the study. |
| **Comment 3** |  |
| Literature Review  Since the authors did not guide the readers to understand the research problem well from the beginning, the contents of this section are also unable to add value to the readers.  It is very obvious that the authors only summarise the past literature on the variables that the authors intend to study. The authors were unable to provide a critical evaluation of these works. Why break the section into High-income, Low-Income Level, Lower Middle-Income Level and Upper Middle-Income Level?  This section is poorly written. The authors failed to integrate arguments into the review. Authors simply summarising their readings. | Thank you for your feedback. We have taken note of your suggestion to make a clearer and more concise explanation. We believe that including the income level is important, since it can impact economic globalization on agriculture value-added. The economic conditions and policies of a country, which are often linked to its income level, can have an impact on how economic globalization affects its agricultural sector. Therefore, by breaking down the analysis based on income levels, we can provide a more nuanced understanding of the economic globalization on agriculture value-added. We have also improved the literature review by bringing relevant literature and critically evaluating. |
| **Comment 4** |  |
| Data and Methodology This section is weakly written. The authors only present the variables intended to study. There is no theoretical framework or hypothesis to support the methods. All the variables and methods are basically formed by the readers without any literature support. | Thank you for your valuable feedback regarding the theoretical framework and hypothesis of our research. We acknowledge the importance of clearly presenting our theoretical framework and hypothesis to support our research methods.  To address this concern, we have added a newly separated paragraph in the literature review section that clearly presents our hypothesis. Additionally, we have included relevant references from reputable sources such as Greene WH. Econometric analysis: Pearson Education India; 2003, Fox J. Applied regression analysis and generalized linear models: Sage Publications; 2015, Neter J, Kutner, M. H., Nachtsheim, C. J., & Wasserman, W. Applied linear statistical models: McGraw-Hill!Irwin; 2005., and Gujarati. Basic Econometrics: Namibia University of Science and Technology; 2004 to support our methodology in the revised manuscript. |
| **Comment 5** |  |
| Results This manuscript only stated the findings without interpretation. | Thank you for your valuable feedback regarding the interpretation of our research findings. We acknowledge the importance of providing a thorough interpretation of our results.  To address this concern, we have included a separate section in the revised manuscript that discusses the interpretations of our findings under policy implications. We appreciate your feedback and are committed to ensuring that our research is presented in a clear and informative manner, with a thorough interpretation of our results. |
| **Comment 6** |  |
| Discussion This section is poorly written. Too much description and not enough analysis. The authors basically cited a lot of literature to end a sentence. | Thank you for your valuable feedback regarding the writing style and analysis of our research. We have carefully considered your comments and have made revisions to the manuscript to address your concerns.  We have streamlined our writing style and reduced the number of citations to provide a more focused and analytical discussion. We appreciate your feedback and are committed to ensuring that our research is presented in a clear, concise, and analytical manner. |
| **Comment 7** |  |
| Policy Implications This section is incompetently written. The authors basically mentioned “the government should …” for all the variables examined in this study. | Thank you for bringing this to our attention. We have taken your feedback seriously and have revised the relevant section accordingly. We appreciate your insights and hope that the revised version meets your expectations. |
| **Comment 8** |  |
| Conclusion The authors only summarise the study. The conclusion is intended to help the reader understand why your research should matter to them after they have finished reading the paper. A conclusion is not merely a summary of your points or a re-statement of your research problem but a synthesis of key points. | Thank you for your suggestion, we have amended the conclusion by synthesising the key points and highlighting the empirical, policy and practical importance of the research. |
| **REVIEWER #4** | |
| **Comment 1** |  |
| The English language needs more work. There are many grammatical and typo mistakes in this manuscript. The paper needs to be edited by a native English speaker. | Noted with thanks. The paper has been revised thoroughly and in-depth copy edit has conducted by an experienced copy-editor. We trust that the revised manuscript is free from any language errors. |
| **Comment 2** |  |
| I noticed that the novelty of this paper is not described in detail. This should be put in the introduction section properly. There is a need to do a more rigorous an asymmetric literature review. It should update literature to current... The authors should clearly mention the literature gap. | Thank you for your valuable feedback regarding the novelty and literature review of our research. We acknowledge the importance of clearly describing the uniqueness of our research and conducting a rigorous literature review.  To address this concern, we have included a separate paragraph in the introduction section, from line 176 to 187, which highlights the research gap and explains how our research contributes to filling this gap. Additionally, we have discussed the literature gap in the revised manuscript, from line 398 to 499. |
| **Comment 3** |  |
| I would like to suggest that authors should update the introduction and results part. Specifically, the latest research trends, and in order to highlight the academic frontier of the research, the references of the recent year need to be referenced. <https://doi.org/10.1016/j.renene.2021.07.014>,  <https://doi.org/10.1002/pa.2712>, <https://doi.org/10.3390/su12072930>, <https://doi.org/10.1016/j.energy.2021.122515>, <https://doi.org/10.1016/j.renene.2021.10.067>, <https://doi.org/10.1016/j.pnucene.2022.104533>, <https://doi.org/10.1007/s13762-022-04638-2>, <https://doi.org/10.1007/s11356-022-23179-2> | Noted with thanks! We revised our citations considering the references suggested by this reviewer and adding new and appropriate past literature.  Citations recommended to be newly added are incorporated in the revised manuscript.  “…Previous investigations revealed that labor force participation has a negative impact on economic growth in Southern Asia, but it has a positive effect in Western Asia. Moreover, the study found a robust and positive relationship between trade openness, human capital, and economic growth”. <https://doi.org/10.3390/su12072930>  This was added in revised manuscript in line 251 to 254.  “Extant empirical literature investigates how AVA in BRICS-T (Brazil, Russia, India, China, South Africa, and Turkey strengthens the potential for the region's ecological footprint to increase and how a one Per cent influence on agriculture raises it by 0.2201 Per cent” <https://doi.org/10.1016/j.renene.2021.07.014>.  This was added in revised manuscript in line 65 to 71.  “…Furthermore, Past study indicates that factors such as AVA, economic growth, non-renewable energy use, and tourism sector expansion have a significant impact on environmental degradation, highlighting their adverse effects on the quality of the environment” <https://doi.org/10.1002/pa.2712>  This was added in revised manuscript in line 68 to 71.  “…The contemporary era of globalization recognizes the significance of financial and natural resources as crucial factors that play a vital role in reducing environmental degradation while simultaneously promoting economic growth “  <https://doi.org/10.1016/j.energy.2021.122515>  This was added in revised manuscript in line 60 to 63. |
| **Comment 4** |  |
| How did the authors get from the theoretical model to the empirical one? Behind the model there need to be a complete and well-thought-out theoretical grounding part of the article shouldn't include any citations or references; rather, it should structured according to the authors' reasoning. The empirical model will come when this part has been completed. | Thank you for your valuable feedback regarding the theoretical and empirical grounding of our research. We have developed a well-thought-out theoretical model and provided a comprehensive discussion of its theoretical foundation in the introduction section, which is supported by evidence from relevant past literature.  To ensure clarity and transparency, we have provided the empirical model at the end of the literature section. The revised manuscript includes these sections from line 392 to 397.  We appreciate your feedback and are committed to ensuring that our research is grounded in a sound theoretical foundation and is transparently reported. |
| **Comment 5** |  |
| The authors should present the main findings in graphical form. It will increase the brevity and more readerships and attract more audience. | Thank you for your valuable suggestion to present the main findings in graphical form. We have considered your suggestion and have revised the manuscript by creating a new table (Table 4) that highlights the main findings. Detail results are now shown in appendix S4. We believe that this table provides a clear and concise overview of the results and will enhance the readability of the paper. |
| **REVIEWER #5** | |
| **Comment 1** |  |
| It is not clear why study period is chosen from 2000 to 2021. | Agreed and thank you for highlighting this point for improvement. Due to data constraints, the study limited its analysis to a maximum of 101 countries and the time period between 2000 and 2021. As a result of data unavailability, observations for other countries and years could not be incorporated into the study.  We have added new section called “Limitation”.  Comment has been incorporated mentioned in line 872 to 874. |
| **Comment 2** |  |
| Model selection is not correct as there could be endogenity problem. Therefore, GMM method is essential. FE and RE models cannot taken seriously in this case. | Thank you for raising the issue of endogeneity in our model selection process. We applied the stepwise method in our analysis. This approach enabled us to systematically test and compare different model specifications while considering the potential endogeneity of certain variables. |
| **Comment 3** |  |
| Choice of variables also not clear. It has to clearly mention for different paragraph for different variables. | Thank you for your feedback regarding the clarity of our variable selection process. We acknowledge the importance of providing clear and detailed explanations for the inclusion of each variable in our analysis.  To address this concern, we have provided a comprehensive discussion of the selected eight variable included in our analysis, outlining their theoretical and empirical relevance in separate paragraphs for each income level and global level. Our aim was to ensure that readers can easily follow our reasoning for including each variable in our model.  We appreciate your feedback and are committed to enhancing the clarity and transparency of our research. |
| **Comment 4** |  |
| Introduction part is not clear why study area is important for the research. Need to rewrite with mentioning the importance and research questions and application of the work. | Thank you for your feedback. We appreciate your constructive criticism and have taken it into consideration. We have revised the introduction to better highlight the importance of the study area and its relevance to the research questions and applications of the work.  Please refer to lines 142-174 for the revised manuscript with track changes. |
| **Comment 5** |  |
| References in the text; mix of number and title of the authors. Should be rewritten as per the journal style. | Thank for noting it down, we have corrected the references as per the PLOS ONE journal style. |
