# Supplementary material for: Impact of economic globalisation on value-added agriculture, globally
Source: PLoS One. 2023 Jul 21;18(7):e0289128. doi: 10.1371/journal.pone.0289128 (PMC10361532; doi:10.1371/journal.pone.0289128)
Supplement: Attachment — Submitted filename: Review report for PONE.docx [file pone.0289128.s008.docx]

Review report for PONE-D-23-05826R1 Impact of Economic Globalisation on Value-Added Agriculture, Globally

**Abstract**

The topic is “Impact of Economic Globalisation on Value-Added Agriculture, Globally”, however the content of the abstract does not reflect the topic. For example, “The results show that agricultural employment significantly impacts the agricultural value added factor globally and across all income levels.” None of it reflect economic globalization. Next, “Also, countries with low and lower middle-income levels significantly affect agricultural value-added due to exchange rates. In comparison, high-income and lower-middle-income levels have an impact due to foreign direct investment. Finally, the upper-middle-income countries have significantly affected agricultural value-added due to agricultural raw materials imports.” The content only reflect the income level of the countries on agriculture. What about economic globalisation? Similarly, “This study confirms that employment in agriculture, exchange rate and foreign direct investments positively impact agriculture value-added on the global level and based on the income level of countries.” Do that employment in agriculture, exchange rate and foreign direct investments reflect anything about economic globalization?

**Introduction**

The flow of this paper does not guide the readers well what are the items that reflect economic globalization. For instance, there are two theories, Heckscher-Ohlin and New Trade theories, how do these theories explaining economic globalization in influencing Agriculture Value Addition (AVA)? Then, the authors introduce trade, Foreign Direct Investment (FDI), Exchange Rate (ER) Agriculture Raw Material Exports (ARME) and Agriculture Raw Material Imports (ARMI). Why discuss about the variables in the introduction? Introduction should consist of these elements.

- General background information
- Specific background information
- A description of the gap in our knowledge that the study was designed to fill
- A statement of study objective, and (optionally) a brief summary of study

Research motivation is missing for this research objective. “This research was conducted globally with four different income levels: high-income, lower- and upper-middle-income countries based on World Bank categorization.”

**Literature Review**

The authors break the section to review article related to different income level. It is inappropriate since the authors did not provide a strong research motivation why there is a need to conduct globally with four different income levels: high-income, lower- and upper-middle-income countries.

**Data and methodology**

Table 1, please show which variables to reflect economic globalization.

Equation 1 to 6, why different region is based on different variables? There is no discussion related to it. It is not correct to present the variables without proper explanation.

Please explain why it is removed? “In the panel data regression, the ARME variable was removed for all countries, the high-income and low-income levels, the ARMI and Trade variables were removed for the lower middle-income level, and the trade variable was released for the upper middle-income level due to changes in the sign of the coefficient values.”

**Results**

Too much figures (1 to 8) but they are not being effectively communicated to the readers. The authors only explaining the figures, but what is the implication and how they contribute to our understanding of the research question? If they are unnecessary, please remove them. This manuscript it too lengthy with too much of unimportant information.

**Discussion**

“However, it has an inverted U-shaped feature in the long-term [68] and supports the present study findings, which significantly impacts FDI on AVA in the high-income level.” From where you come to this interpretation? I do not see your model is examining non-linear impact between the variables.

**Conclusion**

Since the research motivation is not well established from the beginning of the manuscript, it is difficult to convince the readers why this research is matter. Furthermore, the content in the conclusion of this manuscript is too general. It does not succinctly tell the reader how and why it is that what's been presented is significant for practice, policy or further research.
