# Supplementary material for: Impact of economic globalisation on value-added agriculture, globally
Source: PLoS One. 2023 Jul 21;18(7):e0289128. doi: 10.1371/journal.pone.0289128 (PMC10361532; doi:10.1371/journal.pone.0289128)
Supplement: Attachment — Submitted filename: Response to Reviewers.docx [file pone.0289128.s009.docx]

**Point by point response to editor and reviewers**

Dear editor and the reviewers,

We would like to express our profound appreciation to the editor and the reviewers for the valuable comments and suggestions made on our manuscript which were very helpful in revising and improving it.

Please note that the line numbers referred in this document is aligned with the revised manuscript which has track changes.

| **Comment of the Reviewer** | **Comment of the Authors** |
| --- | --- |
| 1.If the authors have adequately addressed your comments raised in a previous round of review and you feel that this manuscript is now acceptable for publication, you may indicate that here to bypass the “Comments to the Author” section, enter your conflict-of-interest statement in the “Confidential to Editor” section, and submit your "Accept" recommendation.  **Reviewer #1: All comments have been addressed.**  **Reviewer #2: All comments have been addressed.**  **Reviewer #3: (No Response)**  **Reviewer #4: All comments have been addressed.**  **Reviewer #5: (No Response)** | Well noted. Thank you for your insightful comment. Your feedback is greatly appreciated and highly valued. |
| 2. Is the manuscript technically sound, and do the data support the conclusions?  The manuscript must describe a technically sound piece of scientific research with data that supports the conclusions. Experiments must have been conducted rigorously, with appropriate controls, replication, and sample sizes. The conclusions must be drawn appropriately based on the data presented.  **Reviewer #1: Yes**  **Reviewer #2: Yes**  **Reviewer #3: No**  **Reviewer #4: Yes**  **Reviewer #5: Partly** | Thank you for your comment; It is highly appreciated. |
| 3. Has the statistical analysis been performed appropriately and rigorously?  **Reviewer #1: Yes**  **Reviewer #2: Yes**  **Reviewer #3: No**  **Reviewer #4: Yes**  **Reviewer #5: No** | Duly Noted. The statistical analysis was expanded thought carefully planned response. |
| 4. Have the authors made all data underlying the findings in their manuscript fully available?  The [PLOS Data policy](http://www.plosone.org/static/policies.action#sharing) requires authors to make all data underlying the findings described in their manuscript fully available without restriction, with rare exceptions (please refer to the Data Availability Statement in the manuscript PDF file). The data should be provided as part of the manuscript or its supporting information, or deposited in a public repository. For example, in addition to summary statistics, the data points behind means, medians, and variance measures should be available. If there are restrictions on publicly sharing data—e.g. participant privacy or use of data from a third party—those must be specified.  **Reviewer #1: Yes**  **Reviewer #2: Yes**  **Reviewer #3: No**  **Reviewer #4: No**  **Reviewer #5: Yes** | Noted with thanks! The authors have ensured that all data underlying the findings are available in the manuscript. |
| 5. Is the manuscript presented in an intelligible fashion and written in standard English?  PLOS ONE does not copyedit accepted manuscripts, so the language in submitted articles must be clear, correct, and unambiguous. Any typographical or grammatical errors should be corrected at revision, so please note any specific errors here.  **Reviewer #1: Yes**  **Reviewer #2: Yes**  **Reviewer #3: No**  **Reviewer #4: Yes**  **Reviewer #5: Yes** | Gratefully received and noted. The help of a qualified language editor has enhanced the language. |
| **REVIEWER 1#** | |
| **Comment 1** |  |
| I have been impressed with your efforts to revise your manuscript and meet the standards of the Plos One journal. The only award you deserve is that this manuscript has been accepted for publication in the Plos One journal. | Thank you very much for your acceptance and positive feedbacks. |
| **REVIEWER 2#** | |
| **Comment 1** |  |
| The revised version is satisfactory, as the authors have incorporated all my comments. The authors have done a commendable job and incorporated all my comments; hence, I don't add any other revisions to them. | Thank you very much for your acceptance and positive feedback. |
| **REVIEWER 3#** | |
| **Comment 1** |  |
| Abstract  The topic is “Impact of Economic Globalisation on Value-Added Agriculture, Globally”, however the content of the abstract does not reflect the topic. For example, “The results show that agricultural employment significantly impacts the agricultural value added factor globally and across all income levels.” None of it reflect economic globalisation.    Next, “Also, countries with low and lower middle-income levels significantly affect agricultural value-added due to exchange rates. In comparison, high-income and lower-middle-income levels have an impact due to foreign direct investment. Finally, the upper-middle-income countries have significantly affected agricultural value-added due to agricultural raw materials imports.” The content only reflect the income level of the countries on agriculture. What about economic globalisation?  Similarly, “This study confirms that employment in agriculture, exchange rate and foreign direct investments positively impact agriculture value-added on the global level and based on the income level of countries.” Do that employment in agriculture, exchange rate and foreign direct investments reflect anything about economic globalisation? | Thank you for your valuable feedback, we appreciate your thorough review of the abstract and your insightful comments. Regarding your concern about the abstract not adequately reflecting the topic of economic globalisation, we would like to clarify that we have identified several proxy variables commonly used in past literature to represent economic globalisation. These variables include fertilizer consumption, employment in agriculture, agriculture raw materials imports and exports, exchange rate, and foreign direct investments.  In our study, we have employed these proxy variables to investigate the impact of economic globalisation on value-added agriculture. Specifically, we examine the relationship between the aforementioned variables, which are key components of economic globalisation, and the agricultural value-added factor at both global level and income levels.  To address your concerns and provide further clarity, we have revised the manuscript accordingly. The revisions can be found in the revised version, specifically from line number 35 to 39.  “The findings of our study reveal that economic globalisation, through various channels such as fertilizer consumption, employment in agriculture, agriculture raw materials export and import, exchange rate and foreign direct investment significantly influences the Agricultural value-added factor globally and across different income levels. Furthermore results show…”  Thank you for your insightful comments. In our study, the main objective was to examine the impact of economic globalisation on agriculture value added globally and across different income levels from 2000 to 2021.  To address your concern regarding the limited focus on economic globalisation, we would like to clarify that we have presented the impact of economic globalisation on both the global level and income level. This comprehensive analysis is supported by relevant data and analysis. We have also provided relevant tables and accompanying analysis (please refer to Table 01) to demonstrate the impact of economic globalisation on agricultural value-added.  In the revised manuscript, we have enhanced the discussion to highlight the impact of economic globalisation on agricultural value-added outcomes across different income levels. This includes elaborating on the mechanisms through which economic globalisation, includes factors such as exchange rates, foreign direct investment, and agricultural raw materials imports has an impact on the agricultural value added.  Thank you once again for your valuable feedback, which has helped us improve the manuscript.  In the literature, several studies have established the relationship between these variables and economic globalisation, highlighting their significance as indicators of economic globalisation. For instance, Anyanwu and Anyanwu (2018) explore the relationship between employment in agriculture and economic globalisation, emphasizing how changes in employment patterns within the agricultural sector can be indicative of economic globalisation [476-500 pp.]. Available from: <https://doi.org/10.18488/journal.8.2018.64.476.500>  Likewise, Altanshagai et al. (2022) demonstrate the impact of exchange rates on economic globalisation, emphasizing how fluctuations in exchange rates can impact trade flows, capital movements, and overall economic interconnectedness [2455 p.]. Available from: <https://doi.org/10.3390/su14042455> .].  Furthermore, Nugroho et al. (2021) examine the role of foreign direct investments in the context of economic globalisation, illustrating how cross-border investments contribute to the integration of economies and the diffusion of technology, knowledge, and managerial practices [e0260043 p.]. Available from: <https://doi.org/10.1371/journal.pone.0260043> .]  These studies, along with others in the field, support the contention that employment in agriculture, exchange rates, and foreign direct investments are closely associated with economic globalisation. We have incorporated this information into our revised manuscript to strengthen our discussion.  Once again, we thank you for your valuable feedback, which has helped us improve the manuscript. |
| **Comment 2** |  |
| Introduction The flow of this paper does not guide the readers well what are the items that reflect economic globalisation. | Thank you for your feedback. We appreciate your input. We would like to address your comment regarding the items that reflect economic globalisation and how they are highlighted in the manuscript.  In our study, we have identified several items that reflect economic globalisation. These items include Fertiliser Consumption, Employment in Agriculture, Agricultural Raw Materials Exports, Agricultural Raw Materials Imports, Trade, Exchange Rate, and Foreign Direct Investment. These variables were carefully selected based on their significance in representing economic globalisation in the context of our study.  To provide clarity and ensure the highlighting of these factors reflecting economic globalisation, we have revised the manuscript accordingly. The relevant paragraphs now specify and discuss the factors reflecting economic globalisation. You can refer to the revised version, specifically from line 51 to 134, to find the highlighted information.  We hope that these revisions address your concerns and provide a clearer understanding of how the items reflecting economic globalisation are incorporated in our study. Thank you once again for your valuable feedback. |
| For instance, there are two theories, Heckscher-Ohlin and New Trade theories, how do these theories explain economic globalisation in influencing Agriculture Value Addition (AVA)? | Thank you for your valuable feedback. We greatly appreciate the recommendation from "Reviewer One," and we have taken it into consideration in our study. In response to this recommendation, we have incorporated the Heckscher-Ohlin theory and the New Trade theory into our analysis.  The Heckscher-Ohlin theory provides valuable insights into how economic globalisation influences Agriculture Value Addition (AVA). This theory explains that economic globalisation impacts AVA through various factors, such as resource endowments, trade patterns, and comparative advantage. By considering these factors, we can better understand the relationship between economic globalisation and AVA.  Similarly, the New Trade theory also plays a significant role in our study. This theory emphasizes the importance of product differentiation, technological advancements, and global value chains in enhancing AVA. By incorporating the concepts of product differentiation and technological advancements, we can gain a deeper understanding of how economic globalisation affects AVA.  By incorporating these theories, we aim to provide a comprehensive analysis of the impact of economic globalisation on AVA. We believe that these theories enhance the theoretical framework of our study and contribute to a more nuanced understanding of the relationship between economic globalisation and AVA.  Once again, we sincerely thank you for your valuable feedback, and we are grateful for the opportunity to incorporate these theories into our study.  We have referenced "Feenstra RC, Taylor AM. International economics (3rd edition): New York: Worth Publishers; 2014" from lines 69 to 78 to support these theories and their relevance to our manuscript. |
| Then, the authors introduce Trade, Foreign Direct Investment (FDI), Exchange Rate (ER) Agriculture Raw Material Exports (ARME) and Agriculture Raw Material Imports (ARMI). Why discuss about the variables in the introduction?  Introduction should consist of these elements.  General background information  Specific background information  A description of the gap in our knowledge that the study was designed to fill.  A statement of study objective, and (optionally) a brief summary of study | Thank you for your feedback. We have included a comprehensive framework in the introduction to provide a contextual understanding of our study on the impact of economic globalisation on Agriculture Value Addition. The discussion of key variables such as trade, Foreign Direct Investment (FDI), Exchange Rate (ER), Agriculture Raw Material Exports (ARME), and Agriculture Raw Material Imports (ARMI), Employment in Agriculture (EA) aims to establish general knowledge about agriculture for non-agricultural sectors.  Well noted.  In general background information included, we included the historical background and basic principles related to the research area from line numbers 51 to 134.  Duly noted, the authors have delved into the specific background of our study from line number 135 to 151.    This research was conducted globally, considering four different income levels, and covering the period from 2000 to 2021. The study addresses crucial factors that contribute to filling research gaps, making it unique. Firstly, the study analyzes the four global and income levels separately, using data from 101 nations. Secondly, it applies a novel evaluation approach using stepwise panel data regression. Lastly, the variables and time frames selected for the analysis differ from those used in previous studies, providing a fresh perspective on the topic. These aspects help fill gaps in the existing research. Following that, we address the research gap in lines number 173-184  The main objective of this study is to examine the impact of economic globalisation on agriculture value addition at both the global level and income levels from 2000 to 2021. (Lines 152-153).  Lastly, the brief summary of this study is as follows. The impact of economic globalisation on agriculture value-added is a critical and underexplored area of research. This study fills this gap by conducting a comprehensive analysis for both globally and income levels: high-income, lower-middle-income, upper-middle-income, and low-income countries. By examining the impact between economic globalisation and agriculture value-added across different income levels, the study provides valuable insights for policymakers, researchers, and stakeholders seeking to enhance agricultural productivity and income generation. This research significantly contributes to the existing literature and offers a solid foundation for evidence-based decision-making (lines 160-168). |
| **Comment 3** | |
| Research motivation is missing for this research objective. “This research was conducted globally with four different income levels: high-income, lower- and upper-middle-income countries based on World Bank categorization.” | Thank you for bringing up this comment. The motivation behind this research stems from the critical and underexplored area of understanding the impact of economic globalization on agriculture value-added at both the global level and across different income levels. The study recognizes the significance of investigating this relationship as it has the potential to inform policymakers, researchers, and stakeholders involved in enhancing agricultural productivity and income generation. You can refer to the specific lines 160 to 168 for further details on the motivation and objectives of our research. |
| **Comment 4** |  |
| Literature Review The authors break the section to review article related to different income level. It is inappropriate since the authors did not provide a strong research motivation why there is a need to conduct globally with four different income levels: high-income, lower- and upper-middle-income countries. | Thank you for your valuable feedback and insightful comments. We divided the literature review based on income levels to ensure a comprehensive analysis of the impact of economic globalisation factors on agriculture value addition globally. This approach enhances the reach and applicability of our research, allowing us to examine the specific dynamics and nuances of the relationship between economic globalisation and agriculture value addition across different income levels. |
| Data and methodology Table 1, please show which variables to reflect economic globalisation.  Equation 1 to 6, why the different region is based on different variables?    There is no discussion related to it. It is not correct to present the variables without proper explanation.  Please explain why it is removed. “In the panel data regression, the ARME variable was removed for all countries, the high-income and low-income levels, the ARMI and Trade variables were removed for the lower middle-income level, and the trade variable was released for the upper middle-income level due to changes in the sign of the coefficient values.” | Thank you for the comment. Further changes were made on Table 1 to which variables reflect economic globalisation.  Equations 1 to 6 were divided based on income levels, rather than different regions. The authors sorted the coefficient values of the fixed effects (FE) and random effects (RE) in descending order for the global and each income level. Variables were added to the regression analysis one by one, and if the direction of the coefficient did not align with previous research findings, those variables were removed from the equation. This approach resulted in different variables being used for different income levels.  The authors provided an explanation from line number from 511 to 527 regarding why different income levels are associated with different variables.  The reason is that during the panel data regression analysis conducted for each income level using the same variables, we observed both positive and negative significant impacts on agricultural value added. However, upon reviewing the relevant literature, we found that the signs of these variables differed from the results obtained in our panel data regression. For instance, previous research indicated a significant impact of ARME on agricultural value-added, whereas our findings showed an insignificant impact. As a result, we made the decision to exclude these variables from the income levels specified in your previous comment. |
| **Comment 5** |  |
| Results Too much figures (1 to 8) but they are not being effectively communicated to the readers.  The authors only explaining the figures, but what is the implication and how they contribute to our understanding of the research question? If they are unnecessary, please remove them. This manuscript it too lengthy with too much of unimportant information. | Thank you for the comment. The figures presented in our research depict the average variations of the dependent and independent variables across different income levels from 2000 to 2021. These figures offer valuable insights into the trends and patterns observed in the data, allowing for a visual representation of the relationships and changes over time.  The figures presented in this study offer a profound understanding of the intricacies of the relationship between income levels and their corresponding effects on the dependent and independent variables between 2000 and 2021. These figures provide valuable insights into the patterns and dynamics of economic performance across different income levels. By visually depicting the data, the figures serve as a powerful tool for observing and interpreting the nuances of economic trends and fluctuations. This information is essential for informed decision-making and strategic planning in various contexts. Please refer to lines 639 to 646 for further details. |
| **Comment 6** |  |
| Discussion “However, it has an inverted U-shaped feature in the long-term [68] and supports the present study findings, which significantly impacts FDI on AVA in the high-income level.” From where you come to this interpretation? I do not see your model is examining non-linear impact between the variables. | Thank you for highlighting the interpretation regarding the inverted U-shaped feature and its impact on FDI and AVA in high-income countries. We would like to clarify that while our specific model may not examine the non-linear relationship between variables, we draw upon relevant literature to support our statement regarding the existence of an inverted U-shaped feature. Specifically, we refer to a study (<https://doi.org/10.3390/su11174620>) that suggests the impact of FDI on AVA in high-income countries follows this particular pattern in the long term. By referencing this literature, we acknowledge the broader research and evidence supporting the presence of the inverted U-shaped relationship between FDI and AVA in high-income countries. |
| **Comment 7** |  |
| Conclusion Since the research motivation is not well established from the beginning of the manuscript, it is difficult to convince the readers why this research is matter.  Furthermore, the content in the conclusion of this manuscript is too general. It does not succinctly tell the reader how and why it is that what's been presented is significant for practice, policy or further research. | Thank you for your feedback. The authors have indeed established research motivation in the introduction section of the manuscript, specifically from line number 160 to 168. This section effectively communicates the importance and relevance of the research, providing a clear understanding of why this study matters in the field.  Thank you for your response. We acknowledge your disagreement with our previous assessment. It is positive to hear that the conclusion provides a concise and informative summary of our research findings. Furthermore, the addition of a section discussing the implications of our findings for practice, policy, and further research enhances the applicability and significance of our study. |
| **REVIEWER 4** | |
| **Comment 1** |  |
| The authors have addressed my comments well. Therefore, this study can be accepted for publication in this journal. | Thank you very much for your accepting the publication of PLOS ONE. |
| **REVIEWER 5** | |
| **Comment 1** |  |
| The primary advantage of stepwise regression is that it's computationally efficient. However, its performance is generally worse than alternative methods. The underlying goal of stepwise regression is, through a series of tests (e.g. F-tests, t-tests) to find a set of independent variables that significantly influence the dependent variable. The best way to deal with endogeneity concerns is through instrumental variables (IV) techniques. The most common IV estimator is Two Stage Least Squares (TSLS). Or GMM method can be used. So still endogeneity problem yet not solved.  I have no problem to use stepwise method. However, to have a robust results GMM approach should be used which will take care endogeneity problem. | Thank you so much for your valuable comment. The authors agreed that the best way to address endogeneity concerns are through instrumental variables (IV) techniques using Two Stage Least Squares (TSLS) or GMM.  The stepwise method is a crucial technique employed in this research. According to Gujarati's book, researchers often utilize the method of stepwise regression to determine the 'best' set of explanatory variables for a regression model. This method involves either introducing the X variables one at a time (stepwise forward regression) or including all possible X variables in multiple regressions and subsequently removing them one at a time (stepwise backward regression). The decision to add or drop a variable is typically based on its contribution to the explained sum of squares (ESS), assessed using the F test. (Gujarati. Basic Econometrics: Namibia University of Science and Technology; 2004.)  According to the stepwise method, the authors have addressed the endogeneity problem effectively. Furthermore, in their future research (refer to lines 783to 786 in the manuscript), the authors plan to enhance the robustness of their findings by incorporating the application of Two Stage Least Squares (TSLS) or Generalized Method of Moments (GMM) as common instrumental variable (IV) estimators. This addition of TSLS or GMM to their methodology further strengthens the validity of their results.  "….When implementing their methodology, the authors can consider employing the Two Stage Least Squares (TSLS) or Generalized Method of Moments (GMM) as widely used instrumental variable (IV) estimators, in addition to the stepwise method." |
