# Supplementary material for: Impact of economic globalisation on value-added agriculture, globally
Source: PLoS One. 2023 Jul 21;18(7):e0289128. doi: 10.1371/journal.pone.0289128 (PMC10361532; doi:10.1371/journal.pone.0289128)
Supplement: Attachment — Submitted filename: Reviewer comment.docx [file pone.0289128.s005.docx]

**Reviewer comment**

The author has presented a well-written article (Impact of Economic Globalisation on Value-Added Agriculture, Globally), detailed, and supported by solid analysis. However, reviewers must make some suggestions to improve this manuscript's quality, including:

1. The manuscript does not have a strong theoretical foundation. I recommend the author add international trade theory and how the research variable represents the theory.
2. The author uses 101 countries and categorized them based on GDP per capita, but the authors need to mention the source of the categorization: What is the World Bank, IMF or other sources?
3. Why do the authors make different models for each country category? This makes the conclusion and policy implications inappropriate. Moreover, the assumption of economic globalization is all countries face the same external conditions so the author needs to make the same model between countries. Literature review and incomplete data cannot be the justification for differences in models between countries.
4. The author is expected to check again Table 3. For example, is it right to choose RE for all countries model? Even though the F-Test and Hausman Test show H_0_ is rejected so the best model is Fixed Effect.
5. The author should also choose only 1 best model (RE or FE) to be displayed in Table 4. In addition, what is meant by numbers in brackets in Table 4? probability value or standard error? If it is the probability: several variables should be significant (p-value is smaller than 0.05).
6. Please the authors to improve discussions, policy implications, and conclusion sections based on the revision of the results section
7. The author needs to add limitations and further research in the conclusion section.
8. Writing references must be fixed to adjust the PLOS ONE journal template
9. The author should use a professional language editor to improve the quality of this manuscript.

Finally, I hope these various suggestions can help you improve this manuscript's quality and can be published in the Plos One journal. Good luck!
